# Minimally invasive sampling to identify leprosy patients with a high bacterial burden in the Union of the Comoros

**DOI:** 10.1371/journal.pntd.0009924

**Published:** 2021-11-10

**Authors:** Sofie Marijke Braet, Anouk van Hooij, Epco Hasker, Erik Fransen, Abdou Wirdane, Abdallah Baco, Saverio Grillone, Nimer Ortuno-Gutierrez, Younoussa Assoumani, Aboubacar Mzembaba, Paul Corstjens, Leen Rigouts, Annemieke Geluk, Bouke Catherine de Jong

**Affiliations:** 1 Institute of Tropical Medicine, Antwerp, Belgium; 2 University of Antwerp, Antwerp, Belgium; 3 Research Foundation Flanders, Brussels, Belgium; 4 Leiden University Medical Center, Leiden, Netherlands; 5 StatUa Center for statistics University of Antwerp, Antwerp, Belgium; 6 Damien Foundation, Brussels, Belgium; 7 National Tuberculosis and Leprosy control Program, Moroni, Union of the Comoros; Emory University, UNITED STATES

## Abstract

The World Health Organization (WHO) endorsed diagnosis of leprosy (also known as Hansen’s disease) entirely based on clinical cardinal signs, without microbiological confirmation, which may lead to late or misdiagnosis. The use of slit skin smears is variable, but lacks sensitivity. In 2017–2018 during the ComLep study, on the island of Anjouan (Union of the Comoros; High priority country according to WHO, 310 patients were diagnosed with leprosy (paucibacillary = 159; multibacillary = 151), of whom 263 were sampled for a skin biopsy and fingerstick blood, and 260 for a minimally-invasive nasal swab. In 74.5% of all skin biopsies and in 15.4% of all nasal swabs, *M*. *leprae* DNA was detected. In 63.1% of fingerstick blood samples, *M*. *leprae* specific antibodies were detected with the quantitative αPGL-I test. Results show a strong correlation of αPGL-I IgM levels in fingerstick blood and RLEP-qPCR positivity of nasal swabs, with the *M*. *leprae* bacterial load measured by RLEP-qPCR of skin biopsies. Patients with a high bacterial load (≥50,000 bacilli in a skin biopsy) can be identified with combination of counting lesions and the αPGL-I test. To our knowledge, this is the first study that compared αPGL-I IgM levels in fingerstick blood with the bacterial load determined by RLEP-qPCR in skin biopsies of leprosy patients. The demonstrated potential of minimally invasive sampling such as fingerstick blood samples to identify high bacterial load persons likely to be accountable for the ongoing transmission, merits further evaluation in follow-up studies.

## Author summary

Leprosy is the oldest infectious disease known to humankind. We still do not succeed in curbing its transmission, with more than 200,000 new patients detected worldwide each year. Identifying persons with a high burden of bacteria is key to curb transmission. To identify these persons, bacteria are counted in invasive and painful samples like slit skin smears and skin biopsies. We evaluated whether we can use less invasive samples, like fingerstick blood or nasal swabs, to determine the bacterial load. We found that the level of antibodies against *M*. *leprae* (αPGL-I IgM) in fingerstick blood correlates well with the bacterial load determined in skin biopsies from the same leprosy patient. Therefore, a high level of antibodies against *M*. *leprae* in fingerstick blood might identify persons who pose a potential risk for transmission of leprosy and could be prioritized for contact screening, which is essential for control of the disease.

## Introduction

According to the World Health Organization (WHO) the global leprosy (also known as Hansen’s disease) prevalence has decreased to <1 patient per 10,000 population since the year 2000, based on which leprosy is eliminated as a public health problem. However, the annual global incidence has stabilized since 2006, with approximately 200,000 new leprosy patients reported worldwide each year [1], often with heterogeneous distribution in high incidence ‘pockets’.

These persistently high incidence areas also occur in settings with solid leprosy control programs, where patients are diagnosed early and treated appropriately with highly effective multidrug therapy. Moreover, in some regions 30% of the leprosy patients occur in children [2], which supports that transmission continues unabatedly. Although transmission pathways of *Mycobacterium leprae* (*M*. *leprae*) are still not fully understood, evidence shows that the main transmission route appears to be through aerosols/droplets to and from the nasal and oral cavities, also skin-to-skin contact and shedding of bacteria into the environment may play a role [3–5]. An infected contact is thought to be genetically predisposed to progress to either the paucibacillary or multibacillary form of the spectrum, although the majority of infected individuals never develop clinically overt leprosy [6]. Progressing to paucibacillary disease (WHO operational classification: ≤5 lesions), is associated with a predominant protective Th1 type response, while multibacillary leprosy (WHO operational classification: >5 lesions) infection links with Th2 type response and high levels of anti-*M*. *leprae* antibodies against phenolic glycolipid I (PGL-I), which are ineffective at controlling this intracellular disease [7]. Untreated multibacillary patients are considered a likely source of transmission, probably even before they develop symptoms [8]. Diagnosis is entirely clinical as stated by the WHO guidelines, relying on the cardinal signs of leprosy. Once a leprosy patient starts multidrug therapy, it is assumed that chances of transmission are drastically reduced since the numbers of viable bacteria are quickly reduced [9].

Since the decreased attention for leprosy in the last century, many clinicians/health care workers lost their acumen to diagnose leprosy, leading to late- and missed diagnoses [10]. Microbiological confirmation, including measurement of the bacterial load, is not standardized nor endorsed by WHO for leprosy diagnosis. The bacterial load can be determined microscopically and by an *M*. *leprae* specific quantitative real-time PCR (qPCR), typically on slit skin smears or skin biopsies which both represent invasive clinical samples. Unfortunately, access to laboratories facilitating molecular techniques tends to be limited in leprosy endemic countries. Therefore, a low complexity lateral flow assay (LFA) utilizing up-converting reporter particles (UCP) was recently developed to quantitatively detect IgM antibodies against the *M*. *leprae* specific PGL-I (αPGL-I) in human serum [11] and fingerstick blood [12], with documented applicability in *M*. *leprae*- and *M*. *lepromatosis*-infected squirrels [13,14]. This particular αPGL-I UCP-LFA on fingerstick blood (further referred to as αPGL-I test) was found to correlate with the bacterial index (BI) as determined by slit skin smear microscopy and qPCR on slit skin smear [12,15]. Molecular tests have greater sensitivity for detection of *M*. *leprae* than microscopy [16]. In 2011, Martinez *et al*. concluded that the qPCR assay targeting a specific repetitive element (RLEP) [17] was more sensitive than qPCR assays using 16rRNA*/sodA/Ag 85B* [18]. In 2018, we resolved that the RLEP-qPCR is also highly specific and that its sensitivity is superior to classical bacillary index (BI) determination on slit skin smear [16]. In 2020, αPGL-I IgM levels were compared to RLEP-qPCR of slit skin smear [19].

The ComLep study is a cross-sectional study conducted in the Comoros on the island of Anjouan where the average annual incidence rate exceeds 7/10.000. Active case finding is in place since 2008 through Mini Leprosy Elimination Campaigns, which consist of outreach skin clinics at the village level, where anyone with skin problems (including leprosy) is invited for a free dermatological consultation and where free treatment is provided for common minor skin ailments. In the present study, we correlated the RLEP-qPCR based bacterial burden in skin biopsies of leprosy patients as reference, with nasal swab RLEP-qPCR and host-based αPGL-I test, aiming for less invasive proxy indicators for the total bacterial burden of leprosy disease.

## Material & methods

### Ethics statement

The protocol from the ComLep study (clinicaltrials.gov: NCT03526718) was approved by the institutional Review Board of ITM, by the Ethical Committee of the University of Antwerp (B300201731571) and by the Ethical Committee on the island of Anjouan. Written informed consent was obtained from each participant or from the parent/guardian of each participant under 18 years of age. For minors aged 12–17 years, additional signed assent from the minor was obtained before participation in the study. Participant were allowed to (selectively) refuse sampling.

### Study participants

From January 2017 until January 2018, on the island of Anjouan (Union of The Comoros) leprosy patients were diagnosed within the ComLep study. Diagnosis was based on the so-called cardinal signs, i.e. a typical hypopigmented patch with loss of sensation and/or enlarged peripheral nerves. Patients with ≤ 5 lesions were classified as paucibacillary and >5 lesions as multibacillary, according to the WHO operational classification. In addition, whether a patient had ≥ 25 lesion was added as a variable, since having ≥25 lesions, will automatically classify the patient as either borderline borderline, borderline lepromatous or lepromatous according the dermatological aspect of the Ridley-Jopling classification. Only patients who provided both a skin biopsy and a fingerstick blood sample were included in this study (S1 Fig). A contra-indication for a skin biopsy was a single lesion occurring in the face. Nasal swabs were collected in addition to these samples. For multibacillary patients who provided a slit skin smear the BI was determined by microscopy.

### Modified Maxwell DNA extraction

The 4 mm skin biopsy and the nasal swab were stored directly after sampling in 1ml of Disolol (ethanol denatured with 1% isopropanol and 1% methyl ethyl ketone) in screw cap vials, which were shipped to ITM. Upon arrival in the laboratory the skin biopsies were manually grinded with mortar and pestle in 1ml PBS. The obtained skin biopsy suspensions and the nasal swab were treated with an inhouse lysis buffer (1.6 M GuHCl, 60 mM Tris pH 7.5, 1% Triton X-100, 60 mM EDTA, Tween-20 10%) followed by DNA extraction using the Maxwell 16 FFPE Tissue LEV DNA Purification Kit, as described by the manufacturer.

### qPCR assay for *M*. *leprae* detection

The *M*. *leprae* bacterial chromosome contains a family of dispersed repeats (RLEP) of variable structure and unknown function. The repetitive RLEP sequence is highly conserved. Thirty-seven copies of RLEP exist in the chromosome, each containing an invariant 545-bp core flanked in some cases by additional segments ranging from 44 to 100 bps. The qPCR detects the *M*. *leprae*- specific RLEP target. The qPCR assay targets 36 out of the 37 RLEP copies present in the *M*. *leprae* genome, yielding a highly sensitive test [17]. This RLEP-qPCR assay also has proven high specificity [16]. RLEP-qPCR was done for each sample in analytical triplicate following published protocols, using the StepOnePlus cycler [17]. To monitor for false negative results we included an internal positive control (Universal Exogenous qPCR Positive Control (Eurogentec, Belgium)) labelled with a different fluorescent probe than the probe detecting the RLEP target, which amplifies independently from the main RLEP-qPCR, to rule out qPCR inhibition in the sample.

Quantification was done by adding to each qPCR run a serial dilution of *M*. *leprae* reference strain NHDP (3x10^6^-30 RLEP copies) (BEI: ref. number 19350). Based on the Cq-value, slope of the regression line and Y-intercept, the StepOnePlus Software v2.3 provides automatically the RLEP copy number per added template as starting quantity (SQ), to determine the amount of mycobacterial DNA present in a sample. Subsequently, the bacterial load (BL) of the samples was calculated by BL  =  (SQ x [volume of DNA extract/volume of template])/36 RLEP copy numbers.

### αPGL-I UCP-LFA

The capillary fingerstick blood collected with a disposable 20 μl Minivette collection tubes (Heparin coated; Sarstedt), was diluted 1:50 by immediate mixing with 980μl assay buffer. The buffer was supplemented with 1% (*v*/v) Triton X-100 (100 mM Tris pH 8, 270 mM NaCl, 1% (w/v) BSA) to lyse blood cells. The diluted fingerstick blood sample (50μl) was flowed on lateral flow strips the same day after transportation to the central laboratory at ambient temperature. The lateral flow strips were transported to LUMC. By using an anti-IgM UCP reporter conjugate, the human αPGL-I IgM antibodies were detected as described in Corstjens et al. (2019) [12]. UCP materials (NaYF_4_:YB3+, Er3+ polyacrylic-acid coated nano-sized particles, 980 nm excitation and 550 nm emission) were obtained from Intelligent Material Solutions, Inc. (Princeton, NJ, USA). A Packard FluoroCount microtiterplate reader compatible with the UCP technology [20], was used to analyse the lateral flow strips. The results were displayed as the ratio value (R) between Test and Flow-Control signal based on relative fluorescence units (RFUs) measured at the respective lines. For this cohort, the αPGL-I UCP-LFA the R-value threshold was set at 0.29 according to and determined as described in previous studies [12].

### Analytical controls

For molecular analyses, on each sampling day, (negative) environmental control swab samples were collected, to rule out contamination during sampling. A positive (suspension of mouse footpad infected with *M*. *leprae* Thai 53) and a negative extraction control (water) were extracted in parallel with the clinical samples in each run to check extraction performance and to rule out contamination during the extraction procedure. An internal qPCR control, a positive and negative (water) DNA qPCR control were run simultaneously with the samples to check performance of the qPCR and to rule out DNA contamination during the qPCR procedure.

For serological analyses, at the start of the study non-endemic—and endemic (non-diseased health care staff) control sera were included for the αPGL-I test.

### Statistical analyses

Data from the assays were log transformed (zero values were replaced by the minimum value for that variable divided by two). Data obtained for the different laboratory assays were evaluated using parametric hypothesis tests. Comparing a quantitative result between the two groups was carried out using the Welch t-test. The difference between months on treatment and the binary outcome of assays, was evaluated with the non-parametric Mann-Whitney U test. The alternative hypothesis stating significant differences between two outcomes was accepted at a significance level of α = .05.

The multiple regression models modelling bacterial load versus αPGL-I R-values used the 10-based logarithm of the bacterial load (determined by RLEP-qPCR) as dependent variable, with the 10-based logarithm of the R-values of αPGL-I test as the independent, continuous variable, and a grouping variable splitting the population into 3 groups: i) patients with a negative nasal swab and less than 25 lesions, ii) patients with either a positive nasal swab or ≥ 25 lesions, iii) patients with both a positive nasal swab and ≥ 25 lesions. To analyse whether the effect of R-value of the αPGL-I test on the bacterial load was uniform across the 3 groups, the interaction between αPGL-I R-values and nasal swab group was tested for significance. The inclusion of the different factors in the multiple regression model was based on a simple linear regression, modelling the effect of each factor on the log10(bacterial load in skin biopsy). Significant factors were entered into a multiple linear regression model (S1 Table). Subsequently, this regression model to predict the number of the bacilli was simplified using stepwise-forward selection based on the Akaike information criterion (AIC).

Patients having ≥50,000 bacilli in a 4mm biopsy were further referred to as high bacterial load (HBL) patients. To evaluate if it was possible to predict the odds of being a HBL-patient, sensitivity and specificity were calculated for the three independent variables (number of lesions, αPGL-I R-value and nasal swab positivity) separately. For the presence of ≥25 lesions, for the nasal swab positivity by RLEP qPCR and for the continuous αPGL-I R-value, a ROC-curve was constructed, and the optimal cut-off value was determined by the Youden index [21], which was 0.81. Subsequently, 2x2 tables were constructed for the predicted versus observed outcome for each binary independent variable. Furthermore, to evaluate whether combining variables would increase the power of predicting the odds of being an HBL-patient, two multiple logistic regressions with presence of ≥25 lesions (yes or no), αPGL-I R-value (as a continuous variable) and nasal positivity (yes or no).

All analyses were conducted with R version 3.5.0 for Windows (The R foundation, Vienna, Austria).

## Results

From January 2017 until January 2018, 310 (paucibacillary = 159; multibacillary = 151) leprosy patients were diagnosed. For 263 (84.8%) patients, sampling was complete (fingerstick blood samples and skin biopsies), with 260 providing a nasal swab from both nostrils instead of a nasopharyngeal swab. Based on clinical examination, 117 patients were classified as paucibacillary and 146 as multibacillary leprosy. Of these, 137 multibacillary patients provided a slit skin smear, of which 62 had a BI >0. At the time of sampling, 53 of the patients (paucibacillary = 18; multibacillary = 35) had received no treatment, versus 210 (paucibacillary = 99; multibacillary = 111) who started their treatment (Table 1). Female patients, patients under 17 years old and patients with no affected nerves are more likely to be paucibacillary (Table 1). All environmental controls tested negative with qPCR, as did the different positive and negative controls included in the DNA extraction and molecular assays, suggesting accurate qPCR results. The endemic and non-endemic controls sera for the αPGL-I test all tested negative.

**Table 1:** Representation of the patients included in this study according to different variables.

	Number of patients				% patients included in the study
**Patients included in the study July 2017-January 2018**	310				
Patients for whom sampling was complete (skin biopsy*, **fingerstick blood)**	263/310				84.8%
**Patients for whom sampling was complete including nasal swab**	260/310				83.9%
**Classification**					
Paucibacillary	117/263				44.5%
Multibacillary	146/263				55.5%
	**Paucibacillary** (%)		**Multibacillary** (%)		**OR (95%CI)**
**Sex**					
Female	60/117	(51.3)	54/146	(37.0)	1.79 (1.09, 2.94)
**Age (years)**					
≤17	75/117	(64.1)	64/146	(43.8)	2.29 (1.39, 3.77)
**Number of lesions**					
≥25	0/117	(0.0)	57/146	(39.0)	NA
**Affected nerves**					
0	49/117	(41.9)	21/146	(14.4)	4.29 (2.38, 7.74)
**Degree of disability**					
0	117/117	(100)	125/146	(85.6)	NA
1			11/146	(7.5)	
2			10/146	(6.8)	
**Treatment at sampling timepoint**					
Treatment started	99/117	(84.6)	111/146	(76.0)	1.73 (0.92, 3.26)

* Incomplete sampling was due to either a single lesion occurring in the face, which was a contra-indication for a skin biopsy, or due to (selective) refusal. OR: odds ratio, 95% CI: 95% confidence interval.

### Determination of bacterial load with RLEP-qPCR in skin biopsies

**Fig 1:**
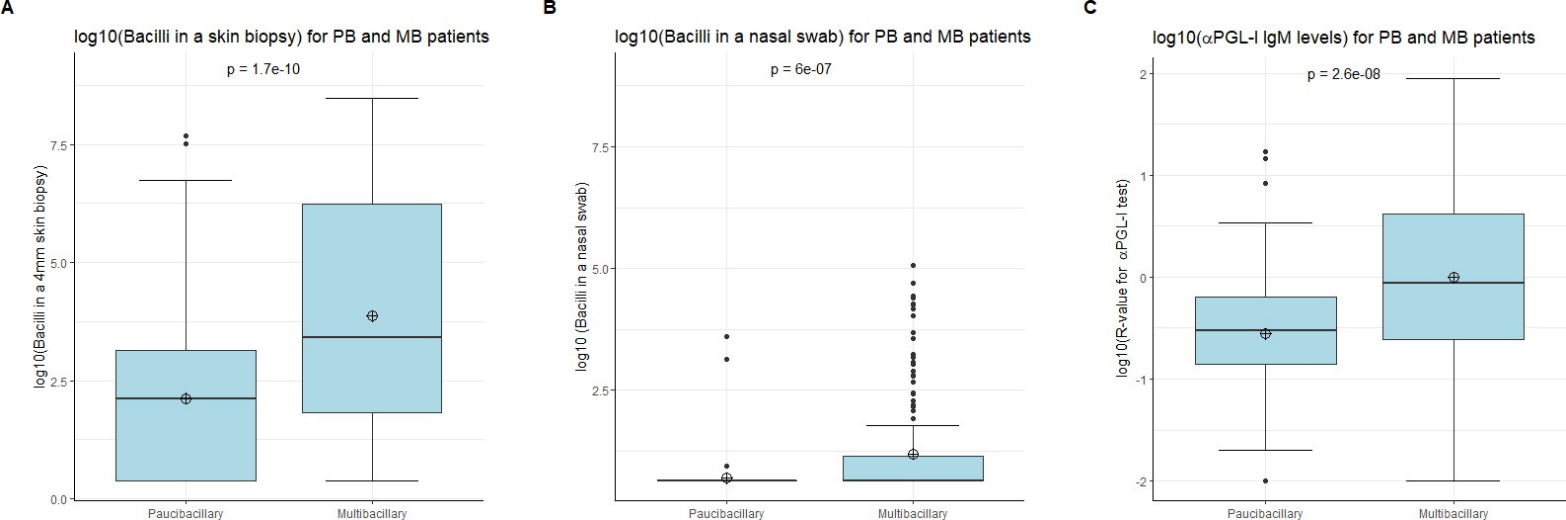
Difference in outcome measure of the assays between paucibacillary and multibacillary patients as per WHO operational classification. (A) Outcome measure of RLEP-qPCR on skin biopsies as determined by the bacterial load in a 4mm skin biopsy of paucibacillary and multibacillary patients as per WHO operational classification. (B) Outcome measure of RLEP-qPCR as determined by the bacterial load in nasal swabs of paucibacillary and multibacillary patients. (C) Outcome measure of the αPGL-I test on fingerstick blood as measured by ratio (R) value, being relative fluorescence units measured at test line divided by the signal measured at the flow-control line of paucibacillary and multibacillary patients.

**Fig 2:**
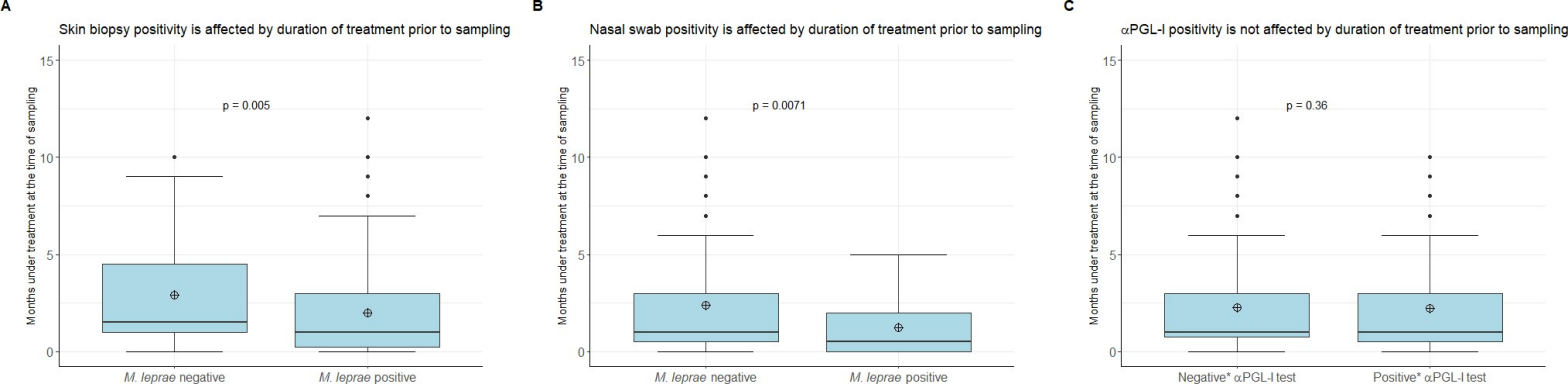
Patients with a negative assay result versus patients with a positive assay result with regard to how long they had been taking multidrug therapy prior to sampling. (A) Patients in whose skin biopsy no M. leprae DNA was detected, had on average been treated longer prior to sampling compared to patients with a detectable amount of M. leprae DNA in their skin biopsy (B) Patients in whose nasal swab no M. leprae DNA was detected, tended to have been treated longer prior to sampling compared to patients with a detectable amount of M. leprae DNA in their nasal swab (C) The presence of systemic αPGL-I is not affected by duration of treatment prior to sampling. R-values for the αPGL-I test on fingerstick blood.*Negative/positive means R-value < infection threshold and R-value ≥ infection threshold respectively.

Of 263 skin biopsies, 196 (74.5%) tested positive for *M*. *leprae* DNA with the RLEP-qPCR (Table 2). Of the paucibacillary patients 79/117 (67.5%) tested positive, versus 117/146 (80.1%) of multibacillary patients (Table 2). As expected, bacterial load detected in skin biopsies was significantly higher in multibacillary patients (median: 2601.5 bacilli; mean of log10(bacilli): 3.872) than in paucibacillary patients (median: 132.0 bacilli; mean of log10(bacilli): 2.12) (Fig 1A; p = 1.7e-10). Of the BI-negative multibacillary patients 51 out of 75 (68.0%) tested positive for the presence of *M*. *leprae* DNA in their skin biopsy (Table 2). In all except one of the 62 BI-positive patients, *M*. *leprae* DNA was detected (98.4%). For 9 multibacillary patients no slit skin smear, and therefore no BI was available (Table 2). There was no significant difference between the bacterial load detected in skin biopsies of paucibacillary and BI-negative multibacillary patients (p = 0.7835). At the time of sampling, patients in whose skin biopsy no *M*. *leprae* DNA was detected, had on average been treated longer compared to patients with a detectable amount of *M*. *leprae* DNA in their skin biopsy (Fig 2A; p = 0.005).

**Table 2:** Laboratory assay results.

	Paucibacillary			Multibacillary									
				BI = 0*			BI>0*			BI = NA*			Total
	N_Pos_	N_Neg_	(%)	N_Pos_	N_Neg_	(%)	N_Pos_	N_Neg_	(%)	N_Pos_	N_Neg_	(%)	(%)
**Skin biopsy RLEP-qPCR**	79	38	**67.5%**	51	24	68.0%	61	1	98.4%	5	4	55.6%	**80.1%**
**Nasal swab RLEP-qPCR**	3	113	**2.6%**	1	73	1.4%	36	25	59.0%	0	9	0.0%	**25.7%**
**Fingerstick blood αPGL-I UCP-LFA**	62	55	**53.0%**	42	33	56.0%	60	2	96.8%	2	7	22.2%	**71.2%**

*BI = bacterial index as determined by microscopy on a skin slit smear; NA = not available, Multibacillary as per WHO clinical definition; Paucibacillary as per WHO clinical definition; N_neg_ = number of negatives for the respective assay; N_pos_ = number of positives for the respective assay.

### Nasal swab positivity determined with RLEP-qPCR

Of 260 nasal swabs, only 40 (15.4%) tested positive for *M*. *leprae* DNA with the RLEP-qPCR (Table 1). The bacterial load detected in the nasal swabs was significantly higher in multibacillary patients than in paucibacillary patients (Fig 1B; p = 6e-07). Only 3 out of the 116 paucibacillary patients (2.6%) tested nasal swab positive, compared to 37/144 (25.7%) multibacillary patients (Table 1). Of the BI-negative multibacillary patients, 1/74 (1.4%) tested positive for *M*. *leprae* DNA in their nasal swab (Table 2), compared to 36/61 (59.0%) of the BI-positive (Table 2). There was no significant difference in bacterial load in nasal swab of paucibacillary and BI-negative multibacillary patients (p = 0.9796). However, patients in whose nasal swab no *M*. *leprae* DNA was detected, tended to have been treated longer prior to sampling compared to patients with a detectable amount of *M*. *leprae* DNA in their nasal swab (Fig 2B; p = 0.0071).

### Level of αPGL-I determined with the αPGL-I test

Fingerstick blood samples of 166/263 (63.1%) patients tested positive for αPGL-I, including 62/117 (53.0%) paucibacillary patients and 104/146 (71.2%) multibacillary patients (Table 2). The level of αPGL-I was significantly higher in multibacillary (median Ratio (R)-value: 0.9; mean log10(R-value): -0.01) than in paucibacillary (median R-value: 0.3; mean log10(R-value): -0.56) patients (Fig 1C; p = 2.6e-08). For the BI-negative multibacillary patients 42/75 (56.0%) tested positive for αPGL-I whereas of the 62 BI-positive multibacillary patients, all but two tested positive for αPGL-I (96.8%). There was no significant difference between the levels of αPGL-I R-value detected in fingerstick blood samples of paucibacillary and BI-negative multibacillary patients (p = 0.3298). Patients with a negative result for the presence of systemic αPGL-I had not been treated longer prior to sampling than patients with a positive result (Fig 2C; p = 0.36).

### Regression model of the αPGL-I test with the bacterial load determined by RLEP-qPCR

**Fig 3:**
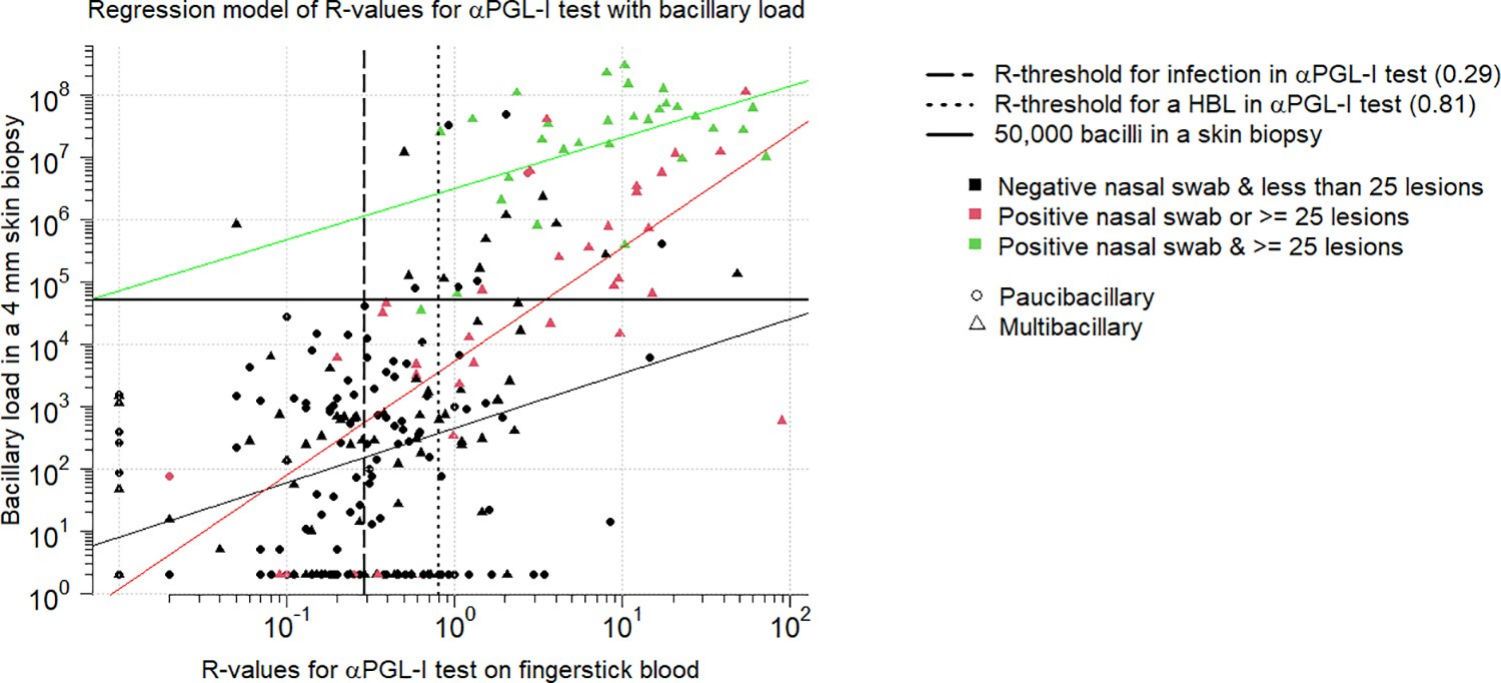
Multiple regression model to estimate the bacterial load in skin biopsies based on the R-value of αPGL-I test, the number of lesions and the nasal swab qPCR result. R-values for the αPGL-I test on fingerstick blood; HBL = high bacterial load (≥50,000 bacilli in a skin biopsy); multibacillary as per WHO operational classification; paucibacillary as per WHO operational classification.

To study the relationship between the αPGL-I IgM levels (measured as R-value of the αPGL-I test) and the bacterial load in skin biopsies, we fitted a multiple linear regression model, including 263 patients (Fig 3). The patients were split in three groups: i) patients with a negative nasal swab and less than 25 lesions, ii) patients with either a positive nasal swab or ≥25 lesions, iii) patients with both a positive nasal swab and ≥ 25 lesions. We tested whether the αPGL-I R-value had an effect on the bacterial load, and if this effect was the same across the 3 groups. We observed an significant relation of the αPGL-I R-value and the bacterial load in a skin biopsy (S2 Table). This relation varied by group (p = 0.01346 for interaction between group and αPGL-I R-values), with the strongest association in the group with either a positive nasal swab or ≥ 25 lesions (Table 3). Exact effect sizes are listed in Table 3. None of the conclusions regarding significance and effect sizes were altered by adding months of treatment prior to sampling to the model.

**Table 3:** Linear relationship between log10(αPGL-I R-value) and log10 (bacterial load in a skin biopsy) for each of the three groups.

	Y-intercept	Slope	Effect Size F-statistic	P-value	Residual standard error	Adjusted R2	Pearson’s r
**Group 1:** < 25 lesions and Neg. nasal swab	2.65 (95%CI: 2.51–2.79)	0.88 (95% CI: 0.71–1.05)	24.4	P = 1.696e-06	1.63 (192 df)	0.11	0.34
**Group 2:** ≥25 lesions or Pos. nasal swab	3.72 (95%CI: 3.46–3.99)	1.83 (95% CI: 1.55–2.10)	40.97	P = 2.609e-07	1.58 (34df)	0.53	0.74
**Group 3:** ≥25 lesions and Pos. nasal swab	6.50 (95%CI: 6.21–6.78)	0.82 (95% CI: 0.55–1.10)	8.543	P = 0.006792	0.85 (28 df)	0.21	0.46

* The y-intercept, slope, effect size, p-value, residuals standard error, adjusted R^2^ and Pearson’s r are given for the linear regression between log10(αPGL-I R-value) and log10 (bacillary load in skin biopsy) for each group

Out of 263 patients, 64 had a bacterial load in a skin biopsy of ≥50,000 bacilli, further referred to as high bacterial load (HBL) patients. Of all the 64 HBL-patients that provided a fingerstick blood sample, 63 tested positive for αPGL-I test (R-value ≥0.29), and 60 of those had a αPGL-I R-value ≥0.81. All HBL-patients except one provided a nasal swab, of which 34 (54.0%) contained *M*. *leprae* DNA. The 29 HBL-patients with a negative result for the nasal swab tended to have taken treatment longer at the time of sampling than the nasal swab positive HBL-patients (p = 0.03006). Of the same 64 HBL-patients, 57 provided a slit skin smear, including 52 (91.2%) with a mean BI-value >0 (Table 4). Out of the 64 HBL-patients, 7 (10.9%) were paucibacillary patients, including one with infiltrated lesions (Table 4).

**Table 4:** Characteristics and treatment of the 64 high bacterial load (HBL) patients.

	Number of HBL-patients	
**WHO operational classification**		
Paucibacillary	7/64	
Multibacillary	57/64	
	**Paucibacillary**	**Multibacillary**
**New case /Relapse**		
New case	7/7	56/57
Relapse/reinfection*	0/7	1/57
**αPGL-I test**		
R-value <0.81	1/7	3/57
R-value ≥0.81	6/7	54/57
**Slit skin smear**		
BI N.A.	7/7	0/57
BI negative	0/7	5/57
BI positive	0/7	52/57
**Number of lesions**		
<25	7/7	15/57
≥25	0/7	42/57
**Infiltrated lesions on clinical exam**		
no	6/7	37/57
yes	1**/7	20/57
**Treatment**		
Paucibacillary treatment	6/7	0/57
Multibacillary treatment	1**/7	57/57
**Treatment follow-up**		
Completed	7/7	50/57
Lost to follow-up	0/7	7/57

R-values for the αPGL-I test on fingerstick blood; BI = bacterial index as determined by microscopic examination of a skin-slit smear; Multibacillary as per WHO operational classification; Paucibacillary as per WHO operational classification

*Relapse: the patient was sampled at a second episode of disease, either relapse or reinfection (indistinguishable in this study).

** One PB patient was treated with MB treatment (12 months), due to the presence of infiltrated lesions. The nasal swab positive paucibacillary HBL-patient is not the paucibacillary HBL-patient with infiltrated lesions.

### Identifying HBL-patients

The separate analysis of the three independent variables indicates that based solely on the clinical feature (≥25 lesions) a HBL-patient can be identified with 65.5% sensitivity and 92.4% specificity. The highest sensitivity (93.8%) for identifying a HBL-patient is obtained at αPGL-I R-value ≥0.81, however with a reduced specificity of 80.9%. The nasal swab result as predictor for HBL has the lowest sensitivity (54.0%) with the highest specificity (97.0%) (Table 5). Combining predictors, two models resulted both in an AUC of 0.93; among these two models we chose the simplest one, which includes having ≥25 lesions and the αPGL-I R-value as predictors, which increases sensitivity with 20% in comparison to solely counting lesions. Nasal swab positivity increased the specificity of HBL patient identification at the cost of sensitivity; overall the addition of nasal swab results did not improve the AUC (p-value = 0.99) (Table 6).

**Table 5:** Evaluation of the independent predictors for being an HBL-patients, with 2X2 tables and sensitivity/specificity.

	Number of lesions		αPGL-I R-value		Nasal swab RLEP-qPCR	
	<25	≥25	<0.81	≥0.81	Negative	Positive
**Non HBL-patients**	184	15	161	38	191	6
**HBL-patients**	22	42	4	60	29	34
	Sensitivity	Specificity	Sensitivity	Specificity	Sensitivity	Specificity
	65.6%	92.4%	93.8%	80.9%	54.0%	97.0%

HBL = high bacterial load (≥50,000 bacilli in a skin biopsy); αPGL-I R-value = ratio (R) value being the relative fluorescence units measured at test line divided by the signal measured at the flow-control line of the αPGL-I test.

**Table 6:** Multiple logistic regression models to predict being an HBL-patient.

Logistic regression	Variables	AUC	95% Confidence interval	Youden index	Sensitivity Youden index	Specificity Youden index
Multiple logistic regression	≥25 lesions and αPGL-I R-value	93.1%	89.4% - 96.8%	170.1	93.7%	77.4%
	≥25 lesions, αPGL-I R-value and nasal swab positivity	93.1%	89.2% - 96.9%	171.6	74.6%	98.0%

αPGL-I R-value = ratio (R) value being the relative fluorescence units measured at test line divided by the signal measured at the flow-control line of the αPGL-I test; AUC = area under the curve

## Discussion

Our findings confirm a strong correlation between αPGL-I IgM levels and the *M*. *leprae* bacterial load as measured by RLEP-qPCR in skin biopsies. To our knowledge this is the first assessment of the correlation of αPGL-I levels with a quantitative molecular measurement in a skin biopsy taken as a proxy for the bacterial load in a leprosy patient. Although a precise determination of the bacterial load by αPGL-I IgM levels is not possible, a range can be estimated. In 2019 Corstjens *et al* [12] demonstrated that the quantitative αPGL-I test results correlated well with the BI of multibacillary patients, and Tio Coma *et al* [19] extended this finding using nasal swab and slit skin smear of contacts and patients in Bangladesh.

The RLEP-qPCR on skin biopsies was able to confirm 74.5% of all clinically diagnosed leprosy patients, 80.1% of the multibacillary patients, and 98.4% of all BI positive patients. The RLEP-qPCR on nasal swab detected only 15.4% of all leprosy patients, 25.7% of the multibacillary patients, and 60.7% of all BI-positive patients, which is in line with previous data in an Asian cohort [19], yet is 41.2% and 49.6% lower for the paucibacillary and multibacillary patients respectively than in the study performed in non-treated patients conducted by Araujo *et al* [22]. The αPGL-I test on fingerstick blood of clinically diagnosed patients was positive in 63.1% of all patients included, 71.2% of the multibacillary patients, and increasing to 96.8% in those patients with a positive BI. These findings are in line with the genetic predisposition for T cell driven paucibacillary or B cell driven multibacillary leprosy. While the αPGL-I test specificity for leprosy could not be estimated in this cohort, given the absence of a large control group of individuals without leprosy, the αPGL-I IgM is acknowledged to be a marker of *M*. *leprae* infection rather than disease. The advantage of using the αPGL-I test on fingerstick blood to support clinical diagnosis, is that this test is minimally invasive, user-friendly and can be performed in remote laboratories such as in the Union of the Comoros, where no specialized facilities are present. Although the RLEP-qPCR on skin biopsies has overall better sensitivity than the αPGL-I test on fingerstick blood and RLEP-qPCR on nasal swab, the downside of using skin biopsies as a confirmation method, is the invasiveness of the sampling, for which local anaesthesia is necessary and a scar remains. Moreover, to avoid false positives, careful processing of the samples in advanced molecular laboratories needs to be ensured.

### Avoiding misclassification using non-invasive sampling

Untreated leprosy, particularly lepromatous cases, can lead to serious irreversible nerve damage, often resulting in significant disfigurement and disabilities. Treatment is usually based on the spectral phenotype of the disease. Leprosy presents as a spectral disease, which is more complicated than paucibacillary/multibacillary determination, as demonstrated by the Ridley-Jopling classification for which histopathology is crucial [23]. The WHO operational classification, based on counting of lesions as a diagnostic method, has its shortcomings. In this study a negative BI distinguished a group of multibacillary patients whose assay results resembled paucibacillary patients. Also, 7 (10.2%) of the 64 HBL-patients (bacterial load in skin biopsy ≥50,000) would classify as paucibacillary patients following WHO operational classification. The leprosy control team decided at time of clinical diagnosis to categorize one of them as an multibacillary because of the presence of infiltrations. A second paucibacillary patient additionally had a positive nasal swab, which may be transient [24]. Hence, fingerstick blood αPGL-I testing in addition to counting lesions seem to improve differentiation within the spectrum of multibacillary leprosy patients. Ultimately, improve classification may guide more appropriate treatment.

### Identification of high risk index cases is a critical knowledge gap

For leprosy it is extremely difficult to identify high risk index cases based on secondary cases as the incubation period of leprosy is exceptionally long (on average 5 years and can take up to 20 years or longer). Identification of high risk index cases is key to curb transmission, and therefore this remains an important knowledge gap, as identified during the COR-NTD conference (National Harbor, 2019). That multibacillary patients are primarily responsible for *M*. *leprae* transmission has been demonstrated several times [25–27] and Sales *et al*. found that patients with a positive BI were four times more likely to transmit the disease to their contact in comparison with multibacillary patients with a negative BI and eight times more likely with a BI>3 [28]. To obtain a BI score, an invasive sample has to be taken. While αPGL-I IgM is acknowledged to be a biomarker of *M*. *leprae* infection rather than disease, our data confirm that in this cohort in the Union of the Comoros high αPGL-I IgM levels are indicative for a higher bacterial load, which is in line with findings of van Hooij *et al*. in 2017 [15], where they demonstrate that αPGL-I IgM levels correlate with BI determined by microscopy. By applying arbitrary thresholds, an αPGL-I R-value of 0.29 could indicate tentative *M*. *leprae* infection, while a αPGL-I threshold of 0.81 (in addition to counting lesions) would allow to identify HBL-patients who may pose an increased risk for transmission. Adding the nasal swab, which may also be transiently positive, does not result in extra power for identifying HBL-patients. In the PEOPLE study (clinicaltrials.gov: NCT03662022) ongoing investigation for (close contact) secondary cases will allow to test the hypothesis that high risk index cases of *M*. *leprae* can be identified with non-invasive sampling. This will help resolve whether they should be prioritized for extensive contact screening beyond the household, to possibly provide prophylactic treatment to these contacts.

### Limitations

Our patients were sampled at variable intervals since the start of treatment limiting our ability to determine diagnostic sensitivity at baseline for the different tests. To control for potential bias, we added this variable to the regression models as a covariate. However, it was nowhere significant, neither in the full model with the interaction between antibodies response and different groups, nor in the linear regression models for the three separate groups (results not shown). None of the conclusions regarding significance and effect sizes were altered by adding months of treatment prior to sampling to the models. Therefore, the month of treatment was not included as a covariate in any of the models, and it is very unlikely that different treatment intervals would bias the results.

In this study we lack a non-exposed population control for the αPGL-I test, although we incorporated (non-)endemic controls sera. Inclusion of more non-diseased endemic controls may allow to identify titers indicative of (multibacillary) disease versus latent infection.

### Way forward

The extension of the ComLep cohort in the ongoing PEOPLE study, including door-to-door screening for leprosy in highly endemic villages allows to identify the best predictors of transmission to close contacts. Additionally, the genotypic comparison of *M*. *leprae* can confirm transmission chains. Correlation with our findings on the bacterial burden may help identify high risk index cases and their characteristics, and allow us to test whether leprosy patients with higher outcome in the αPGL-I test, e.g. αPGL-I test R-value above 0.81, have significantly more secondary cases than patients with lower αPGL-I levels in a certain time frame. The ongoing adaptation of the αPGL-I, full integration of the test in an individually wrapped cassette and improved availability of readers will facilitate actual Point-of-Care testing.

In conclusion, improved approaches for microbiological confirmation of *M*. *leprae* utilizing less invasive sampling are desired and shown here to be feasible. The bacterial load is not routinely measured for leprosy patients, although it is a known correlate of infectiousness [28]. Counting the number of lesions together with the quantitative αPGL-I IgM levels can be used as a proxy for the bacterial load in leprosy patients and to better classify patients along the clinical leprosy spectrum. Ongoing studies (i.e. PEOPLE study) are expected to provide further evidence whether counting lesions and αPGL-I test on fingerstick blood can identify high risk index cases, who can then be prioritized for extensive contact screening beyond the household.

## Supporting information

S1 Fig Patient flowchart. PB = paucibacillary according to the operational WHO classification; *MB = multibacillary according to the operational WHO classification.
(DOCX)

S1 Table Simple linear regression analysis. The inclusion of the different factors in the multiple regression model was based on a univariable analysis for each of the factors, estimating the influence of a factor to the bacillar load in the skin biopsy.
(DOCX)

S2 Table Coefficients for the multiple logistic regression, showing no signs of multicollinearity. (DOCX)

S3 Table Descriptive statistics for each assay per operational classification. PB = Paucibacillary operational WHO classification; MB = Multibacillary operational WHO classification.
(DOCX)
