# Supplementary material for: Minimally invasive sampling to identify leprosy patients with a high bacterial burden in the Union of the Comoros
Source: PLoS Negl Trop Dis. 2021 Nov 10;15(11):e0009924. doi: 10.1371/journal.pntd.0009924 (PMC8580230; doi:10.1371/journal.pntd.0009924)
Supplement: S1 Fig — PB = paucibacillary according to the operational WHO classification; *MB = multibacillary according to the operational WHO classification. (DOCX) [file pntd.0009924.s001.docx]

**S1 Figure: Patient flowchart**

**
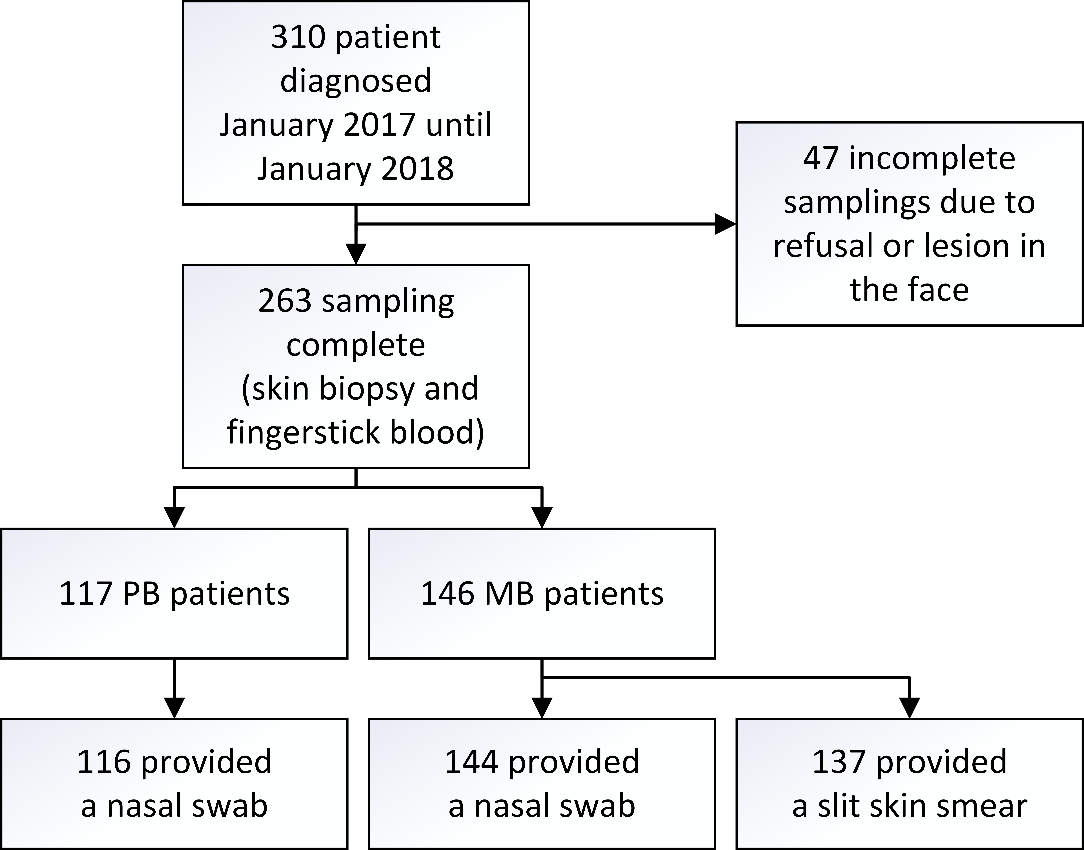
**

*PB= paucibacillary according to the operational WHO classification; *MB= multibacillary according to the operational WHO classification
