# Supplementary material for: Minimally invasive sampling to identify leprosy patients with a high bacterial burden in the Union of the Comoros
Source: PLoS Negl Trop Dis. 2021 Nov 10;15(11):e0009924. doi: 10.1371/journal.pntd.0009924 (PMC8580230; doi:10.1371/journal.pntd.0009924)
Supplement: S1 Table — The inclusion of the different factors in the multiple regression model was based on a univariable analysis for each of the factors, estimating the influence of a factor to the bacillar load in the skin biopsy. (DOCX) [file pntd.0009924.s002.docx]

**S1 Table. Simple linear regression analysis.**

| **Factor** | | **Significance** | **Inclusion** |
| --- | --- | --- | --- |
| **αPGL-I UCP-LFA test value** | | P<0.001*** | Inclusion |
| **Number of lesion (≤or>25 lesions)** | | P<0.001*** | Inclusion |
| **Nasal swab positivity** | | P<0.001*** | Inclusion |
| **Nerves affected** | | P<0.05(.) | Not included |
|  | Painful nerves | P<0.01* | Not included |
|  | Hypertrophic nerves | P<0.01* | Not included |
| **Plaques** | | P>0.05 | Not included |
| **Nodules** | | P<0.001*** | Not included |
| **Sensitivity loss** | | P>0.1 | Not included |

The inclusion of the different factors in the multiple regression model was based on a univariable analysis for each of the factors, estimating the influence of a factor to the bacillary load in the skin biopsy.
