# Supplementary material for: Minimally invasive sampling to identify leprosy patients with a high bacterial burden in the Union of the Comoros
Source: PLoS Negl Trop Dis. 2021 Nov 10;15(11):e0009924. doi: 10.1371/journal.pntd.0009924 (PMC8580230; doi:10.1371/journal.pntd.0009924)
Supplement: S2 Table — (DOCX) [file pntd.0009924.s003.docx]

**S2 Table. Coefficients for the multiple logistic regression, showing no signs of multicollinearity**

| Coefficients: | | **Estimate** | **Standard Error** | **t-value** | **Pr(>\|t\|)** | |
| --- | --- | --- | --- | --- | --- | --- |
|  | intercept | 2.7711 | 0.1354 | 20.461 | | <2e-16*** |
|  | Log10(αPGL-1 R-value) | 1.1077 | 0.1415 | 7.828 | | 1.31e-13*** |
|  | **Group 2:** $\geq$25 lesions or Pos. nasal swab | 1.1710 | 0.3090 | 3.790 | | 0.000188*** |
|  | **Group 3:** $\geq$25 lesions and Pos. nasal swab | 3.4746 | 0.3677 | 9.449 | | <2e-16*** |
|  | **F-statistic**  177.5 (256 df) | **p-value**:  < 2.2e-16 | **Multiple R-squared**: 0.5792, | **Adjusted R-squared**:  0.5743 | | **Residual standard error**: 1.574 (256 df) |
