# Supplementary material for: Minimally invasive sampling to identify leprosy patients with a high bacterial burden in the Union of the Comoros
Source: PLoS Negl Trop Dis. 2021 Nov 10;15(11):e0009924. doi: 10.1371/journal.pntd.0009924 (PMC8580230; doi:10.1371/journal.pntd.0009924)
Supplement: S3 Table — PB = Paucibacillary operational WHO classification; MB = Multibacillary operational WHO classification. (DOCX) [file pntd.0009924.s004.docx]

**S3 Table: Descriptive statistics for each assay per operational classification**

|  | **Log10(Bacilli in skin biopsy)** | | **Log10(bacilli in nasal swab)** | | **Log10(αPGL-I R-value)** | |
| --- | --- | --- | --- | --- | --- | --- |
|  | **PB** | **MB** | **PB** | **MB** | **PB** | **MB** |
| **Mean** | 2.12 | 3.87 | 0.691 | 1.19 | -0.557 | -0.00598 |
| **Median** | 2.12 | 3.42 | 0.641 | 0.641 | -0.523 | -0.0605 |
| **Interquartile range** | 2.75 | 4.43 | 0 | 0.497 | 0.660 | 1.23 |
|  | **Bacilli in skin biopsy** | | **Bacilli in nasal swab** | | **αPGL-I R-value** | |
|  | **PB** | **MB** | **PB** | **MB** | **PB** | **MB** |
| **Mean** | 744633 | 12188796 | 46.6 | 2054 | 0.815 | 6.18 |
| **Median** | 132 | 2602 | 0 | 0 | 0.3 | 0.87 |
| **Interquartile range** | 1347 | 1808697 | 0 | 13.8 | 0.5 | 3.90 |
|  | **Months of treatment** | | | | | |
|  | **Skin biopsy RLEP-qPCR negative** | **Skin biopsy RLEP-qPCR positive** | **Nasal swab RLEP-qPCR negative** | **Nasal swab RLEP-qPCR positive** | **αPGL-I negative** | **αPGL-I negative** |
| **Mean** | 2.92 | 2.01 | 2.39 | 1.24 | 2.22 | 2.26 |
| **Median** | 1.5 | 1 | 1 | 0.5 | 1 | 1 |
| **Interquartile range** | 3.5 | 2.75 | 2.5 | 2 | 2.25 | 2.5 |

**PB= Paucibacillary operational WHO classification; MB=Multibacillary operational WHO classification*
